# Editorial: Nanofertilizers and abiotic stress tolerance in plants

**DOI:** 10.3389/fpls.2023.1154113

**Published:** 2023-04-06

**Authors:** Heba M. M. Abdel-Aziz, Adalberto Benavides-Mendoza, Muhammad Rizwan, Mahmoud F. Seleiman

**Affiliations:** ^1^ Botany Department, Faculty of Science, Mansoura University, Mansoura, Egypt; ^2^ Department of Horticulture, Autonomous Agrarian University Antonio Narro, Saltillo, Mexico; ^3^ Department of Environmental Sciences, Government College University Faisalabad, Faisalabad, Pakistan; ^4^ Plant Production Department, College of Food and Agriculture Sciences, King Saud University, Riyadh, Saudi Arabia; ^5^ Department of Crop Sciences, Faculty of Agriculture, Menoufia University, Shibin El-Kom, Egypt

**Keywords:** nanomaterials, nanoparticles, biostimulants, abiotic stressors, sustainable production, climatic change

Numerous environmental factors have a negative impact on the physiology and yields of plants (Seleiman et al., 2020; Rasheed et al., 2021; Seleiman et al., 2021b; Seleiman et al., 2021c; Sharma et al., 2022; Zandalinas and Mittler, 2022). A number of studies have uncovered plant genes, proteins, and metabolites that govern stress responses (Mittler et al., 2022; Yang et al., 2022; Zhan et al., 2022). Several of these compounds [such as plant growth regulators (Wang and Komatsu, 2022), and amino acids (Zhao et al., 2022)] were utilized to enhance plant resistance to abiotic stressors (Wang and Komatsu, 2022; Zhao et al., 2022). In recent years, nanotechnology has emerged as a viable platform in the era of agriculture, attracting the interest of researchers from a variety of disciplines, particularly for resolving challenges associated with abiotic stresses to assure agricultural sustainability (Saxena et al., 2016; Das and Das, 2018; Seleiman et al., 2021a). Nanoparticles (NPs) are gaining great importance in the field of abiotic stress research because of their unique physio-chemical features, such as extraordinary stability within cells, tiny size (1-100 nm), higher surface area, and higher reactivity (Nejatzadeh, 2021; Elshayb et al., 2022a; Elshayb et al., 2022b; Al-Selwey et al., 2023). It is generally known that NPs dosages can have both beneficial and harmful biological consequences in humans, animals, plants and microorganisms. Nanoparticles caused oxidative damage to biomolecules at high concentrations, which can result in plant cell death. Notwithstanding, at low nanomolar concentrations, NPs operate as crucial biostimulants or regulators of plant growth and development (Nejatzadeh, 2021). The application of NPs improves plant stress resistance by enhancing reactive oxygen species (ROS) detoxification through increasing antioxidant enzyme activities in plants (Jalil and Ansari, 2019; Kumari et al., 2022). Therefore, this Research Topic is aligned with current research trends and provides an update on the advances in nano-fertilizers and abiotic stress tolerance approaches for better understanding plant responses to abiotic stresses. Here, we highlighted some of the topics from the following contributions.

Three articles focused on providing comprehensive reviews of the role of NPs utilized to tolerate different abiotic stresses in plants. In the review by Manzoor et al., these authors looked at the use of different NPs against four abiotic stresses including heavy metals, drought, salinity, and heat stress. This review has demonstrated the ability of NPs to protect crop plants from various abiotic stresses and the methods by which NPs may accumulate in plants. The application of NPs dramatically enhanced the abiotic stress tolerance of plants by enhancing cellular antioxidants, nutrient absorption, photosynthetic efficiency, and biochemical/molecular mechanism regulation. Although nano-fertilizers provide a cost-effective method for enhancing the abiotic stress tolerance of plants by supplying necessary nutrients, their widespread use has raised concerns about their potentially harmful impacts on the environment. Aguirre-Becerra et al. reviewed the use of NPs against different abiotic stresses: drought and salinity stresses, temperature stress, soil contamination with trace metals, and light stress. This review provides an overview of the effect of nanomaterials (NMs) on the abiotic stress resistance of plants. Implementing NMs and NPs is one of the primary solutions advocated for substituting traditional fertilizers, herbicides, and pesticides in agriculture and improving the influence of growth regulators and plant defenses. This relatively new method produces various results depending on the crop, NM content, application type, and application frequency. The specific effect of each NP is determined by its size, surface area, thermal stability, crystallinity, concentration, electromagnetic-optical properties, and origin. Biosynthesis of NPs implementing bacteria, fungi, and plant extracts resulted in enhanced plant resistance to abiotic stress and decreased phytotoxicity. In addition, the nutrient diversity, regulated release, and rapid absorption of NPs make them viable tools for the agri-food industry, resulting in increased crop growth and yield and accelerated germination processes. In the review by El-Saadony et al., the authors provide an overview of the implementation of NPs in abiotic stress tolerance (salinity, drought, and heavy metals) with special reference to Si NPs, Ag NPs, TiO_2_ NPs, and Zn NPs. By activating plant defense systems via the stimulation of reactive oxygen species (ROS) scavenging system, NPs, such as TiO_2_, SiO_2_, and Ag NPs, can minimize the deleterious impacts of abiotic stress. Due to their small size, NPs can also easily permeate plant tissues, where they exert a favorable influence on plant morphological, physiological, and biochemical processes, enhance plant development, and boost crop output in plants subjected to a variety of abiotic challenges. In addition, the vast surface area of NPs enhances the absorption and delivery of numerous specific nutrients.

Three articles discussed the potential of silicon NPs in alleviating different abiotic stresses. Verma et al. reviewed the role of Si NPs in alleviating drought stress. It has been determined that Si NPs play an important role in agroecosystems and enhance stress resistance qualities for future sustainable agriculture. The paper by Riaz et al. examined the effect of nanosilicon on the regulation of ascorbate-glutathione contents, antioxidant defense system, and growth of copper-stressed wheat seedlings. Si NPs can relieve Cu^2+^ stress in wheat seedlings, according to the study’s findings. This research may aid in increasing wheat yield in Cu^2+^-contaminated soils. Desoky et al. examined the integrated application of bacterial carbonate precipitation and Si NPs and found that they enhance the productivity, physiological attributes, and antioxidant defenses of wheat under semiarid conditions. This treatment significantly activated the antioxidant defense system to minimize the detrimental effects of oxidative stress, hence boosting tolerance and enhancing wheat plant output in sandy soils under semiarid climatic conditions.

The study by Dola et al. showed that nanoiron oxide accelerated the growth, yield, and quality of *Glycine max* seeds in water deficit conditions. The results indicated that exogenous foliar sprays were more effective than other treatments in enhancing drought tolerance and can be utilized to reduce productivity losses caused by drought stress in soybean-growing areas. Gao et al. showed that zinc oxide NPs improved the lettuce plant tolerance to cadmium by stimulating antioxidant defense, enhancing lignin content and reducing metal accumulation and translocation.

## Concluding remarks and future perspectives

In summary, the work presented in this Research Topic documents showed how different types of NPs could be effectively applied to mitigate abiotic stresses and improve growth and productivity of different plants. Nanoparticles can activate ROS-scavenging system (compounds and enzymes) synthesis alongside other metabolic processes that support plants to tolerate different environmental stresses. In spite of this, the applications of NPs in crop enhancement and sustainable agriculture are still in their earliest years, and the existing research in the subject is limited and inconsistent. In order to prevent the negative impacts of NPs on ecosystems and crops, it is necessary to carry out further research into the following issues: a) the response of nanoparticles with plants and metabolic processes at the molecular and cellular levels, as well as the optimization of nanoparticles’ size and concentration prior to field application; b) the impacts of NPs and their potential toxicity in various plant species; c) the influence of NPs on gene regulation and expression in plants subjected to diverse abiotic stressors; d) the behaviour and destination of NPs in plants and the environment; e) the influence of soil characteristics and plant species on the efficacy of NPs; (f) the classification of NPs as stress in activators; and (g) the combined impact of NPs with other active components and biotic stressors in plants.

## Author contributions

All authors listed have made a substantial, direct, and intellectual contribution to the work and approved it for publication.
